# Quantitative US fat fraction for noninvasive assessment of hepatic steatosis in suspected metabolic-associated fatty liver disease

**DOI:** 10.1186/s13244-024-01728-2

**Published:** 2024-06-21

**Authors:** Haohao Yin, Yunling Fan, Jifeng Yu, Bing Xiong, Boyang Zhou, Yikang Sun, Lifan Wang, Yuli Zhu, Huixiong Xu

**Affiliations:** 1 Department of Ultrasound, Zhongshan Hospital, Institute of Ultrasound in Medicine and Engineering, Fudan University, Shanghai, 200032 China; 2 Shanghai Institute of Medical Imaging, Fudan University, Shanghai, 200032 China

**Keywords:** Metabolic-associated fatty liver disease, Ultrasonography, Proton magnetic resonance spectroscopy

## Abstract

**Objectives:**

To evaluate the agreement between quantitative ultrasound system fat fraction (USFF) and proton magnetic resonance spectroscopy (^1^H-MRS) and the diagnostic value of USFF in assessing metabolic-associated fatty liver disease (MAFLD).

**Methods:**

The participants with or suspected of MAFLD were prospectively recruited and underwent ^1^H-MRS, USFF, and controlled attenuation parameter (CAP) measurements. The correlation between USFF and ^1^H-MRS was assessed using Pearson correlation coefficients. The USFF diagnostic performance for different grades of steatosis was evaluated using receiver operating characteristic curve analysis (ROC) and was compared with CAP, visual hepatic steatosis grade (VHSG).

**Results:**

A total of 113 participants (mean age 44.79 years ± 13.56 (SD); 71 males) were enrolled, of whom 98 (86.73%) had hepatic steatosis (^1^H-MRS ≥ 5.56%). USFF showed a good correlation (Pearson *r* = 0.76) with ^1^H-MRS and showed a linear relationship, which was superior to the correlation between CAP and ^1^H-MRS (Pearson *r* = 0.61). The USFF provided high diagnostic performance for different grades of hepatic steatosis, with ROC from 0.84 to 0.98, and the diagnostic performance was better than that of the CAP and the VHSG. The cut-off values of the USFF were different for various grades of steatosis, and the cut-off values for S1, S2, and S3 were 12.01%, 19.98%, and 22.22%, respectively.

**Conclusions:**

There was a good correlation between USFF and ^1^H-MRS. Meanwhile, USFF had good diagnostic performance for hepatic steatosis and was superior to CAP and VHSG. USFF represents a superior method for noninvasive quantitative assessment of MAFLD.

**Critical relevance statement:**

Quantitative ultrasound system fat fraction (USFF) accurately assesses liver fat content and has a good correlation with magnetic resonance spectroscopy (^1^H-MRS) for the assessment of metabolic-associated fatty liver disease (MAFLD), as well as for providing an accurate quantitative assessment of hepatic steatosis.

**Key Points:**

- Current diagnostic and monitoring modalities for metabolic-associated fatty liver disease have limitations.
- USFF correlated well with ^1^H-MRS and was superior to the CAP.
- USFF has good diagnostic performance for steatosis, superior to CAP and VHSG.

**Graphical Abstract:**

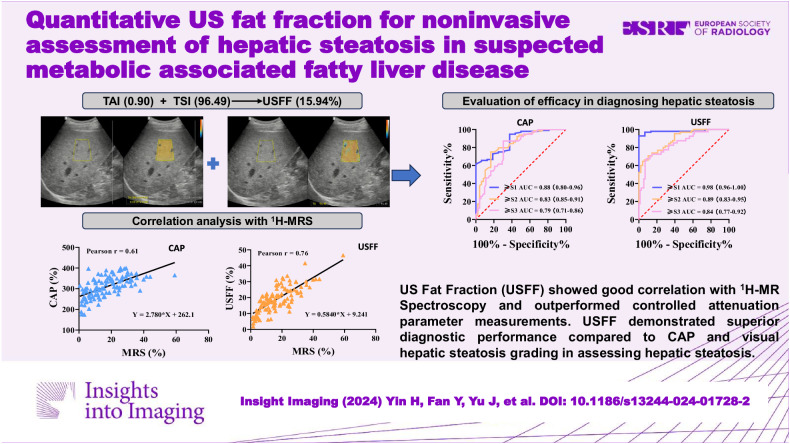

## Introduction

Metabolism-associated fatty liver disease (MAFLD) is a prevalent condition affecting over one-third of the world’s population [1]. Hepatic steatosis, also known as fatty liver disease, is a condition characterized by the accumulation of excess fat in the liver. It can be diagnosed through histological examination of liver biopsy specimens, blood biomarkers or scores, or imaging modalities such as ultrasound or magnetic resonance imaging (MRI). Hepatic steatosis is a common finding in individuals with overweight or obese (body mass index, BMI > 25 kg/m^2^), and metabolic dysfunction (including hypertriglyceridemia, hypercholesterolemia, increased waist circumference, insulin resistance, and systemic hypertension) [2]. MAFLD is not a single lesion, but rather a spectrum of progressive stages of liver disease, starting with simple steatosis and progressing to steatohepatitis, liver fibrosis, cirrhosis, and hepatocellular carcinoma (HCC) [3]. There are several important complications associated with MAFLD that can lead to increased mortality including cardiovascular disease (CVD), non-hepatic malignancies, lung disease, chronic kidney disease (CKD), cognitive impairment, and complications of type 2 diabetes mellitus (T2DM). CVD is the leading cause of death in these patients [4]. Due to the global increase in obesity and diabetes mellitus, the prevalence of MAFLD and its complications are on the rise [5]. Therefore, it is crucial to accurately identify hepatic steatosis in the clinical setting for timely diagnosis and treatment.

While liver biopsy remains the current gold standard for diagnosing MAFLD, it is an invasive procedure that carries risks of complications like bleeding and infection [6]. Moreover, the small amount of liver tissue obtained during a biopsy (1/50,000 of the liver) can result in sampling variation, making histological evaluation challenging [7]. Therefore, there is a critical need for multiple assessment methods, particularly noninvasive ones, to accurately diagnose and monitor hepatic steatosis [8]. MRI-based methods, including MRI proton density fat fraction (MRI-PDFF) and proton magnetic resonance spectroscopy (^1^H-MRS), can accurately and reproducibly assess hepatic steatosis with an area under the curve (AUC) for identifying hepatic steatosis as high as 0.99 [9], but these are not widely used due to their high cost and low accessibility [10, 11]. Ideally, a modality should be noninvasive, accurate, cost-effective, and provide a point of care and clinical assessment of the degree of hepatic steatosis in patients with MAFLD [12].

Conventional ultrasound (US) is the most common imaging modality used to assess hepatic steatosis because of its safety, noninvasiveness, and low cost. Moreover, US techniques demonstrated good reproducibility [13]. However, conventional US has limitations in terms of quantitative accuracy and reproducibility due to operator dependence [14]. Furthermore, accurately grading the degree of steatosis in conventional US is challenging, which may lead to underdiagnosis of low-grade steatosis [15]. Therefore, multiple methods have been investigated to extract quantitative information from the US to improve steatosis screening [16–21], each with advantages and disadvantages. For example, steatosis can be assessed by the hepatorenal index, which is a computer-assisted measurement of the hepatic and renal ultrasound echo intensity ratio (H/R) to measure the degree of hepatic steatosis [22]. However, displaying the right kidney and liver on the same ultrasound image can be challenging for inexperienced radiologists, particularly when examining obese patients or those who have had renal surgery resulting in renal morphological changes or split liver transplantation. This can make measuring the H/R ratio difficult [18]. The controlled attenuation parameter (CAP) method used by FibroScan® is the first approved and most widely used attenuation-based technique [19, 23]. The hepatic steatosis classification correlates with the measurement of the US attenuation signal in dB/m by a series of algorithms ranging from 100–400 dB/m, known as CAP [24]. The diagnostic accuracy of CAP has been extensively validated [25], with more than 10% of steatosis being differentiated by CAP. However, CAP has some disadvantages. It does not allow for the simultaneous assessment of hepatic morphological changes and has a high rate of measurement failure, making it ineffective in patients with a BMI [26]. To address the limitations of CAP in visualizing the liver, several techniques have been developed. These include attenuation imaging (ATI) [27], attenuation factor™ (ATT), ultrasound-guided attenuation parameter (UGAP) [14], tissue attenuation imaging (TAI), and tissue scattering distribution imaging (TSI) [11]. These noninvasive quantitative techniques may be appropriate for large-scale screening and for repeated measurements of liver fat content during longitudinal follow-up. This allows for dynamic monitoring of disease progression and shows promising potential for clinical use.

Quantitative ultrasound (QUS) techniques such as TAI and TSI have been applied to quantify liver fat with high intra- and intergroup reproducibility and agreement [28–30]. Meanwhile, a fat fraction (FF) estimator for QUS has been developed (USFF) that is able to convert directly from QUS to the corresponding liver fat fraction [31]. Studies have shown that ^1^H-MRS is more effective than conventional US in detecting various grades of hepatic steatosis, particularly mild hepatic steatosis [32]. The aim of this study is to explore the correlation between USFF and ^1^H-MRS, and to evaluate the advantages and disadvantages of USFF compared to conventional visual hepatic steatosis grade (VHSG) and CAP in diagnosing different grades of hepatic steatosis. Additionally, reference thresholds for different grades of steatosis will be provided.

## Materials and methods

This prospective, single-institution, cross-sectional study was conducted in Fudan University Zhongshan Hospital. Our institutional review board approved this prospective study (approval number: B2021-0921R). All participants provided written informed consent. The authors had control over the data and information submitted for publication. The study was conducted in accordance with the Declaration of Helsinki.

### Study participants and design

**Fig. 1:**
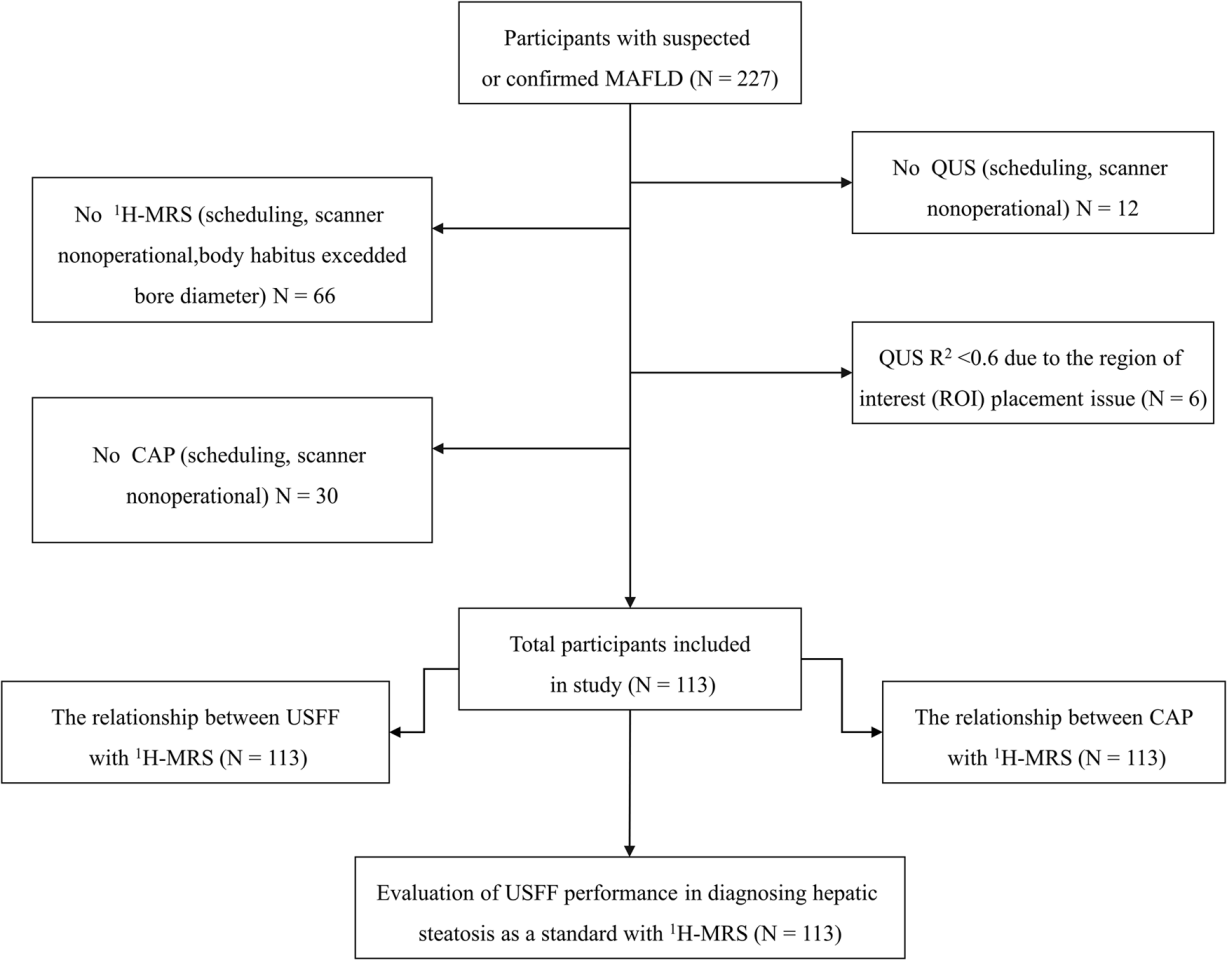
Flow chart of participants included and excluded from this study

Study participants were recruited consecutively and prospectively between August 2022 and May 2023 by one radiologist (Y.L.Z. with 10 years of abdominal ultrasound experience) from the Center for the Study of MAFLD at Zhongshan Hospital, Fudan University. The diagnostic criteria for MAFLD followed the international expert consensus and clinical practice guidelines [2, 33]. Inclusion criteria for this study were: (a) age > 18 years; (b) clinical suspicion of MAFLD, i.e., the existence of fatty liver based on imaging or even blood biomarkers/score, like fatty liver index (FLI) and one of the following three conditions: overweight/obesity (BMI ≥ 23 kg/m^2^), type 2 diabetes mellitus, and metabolic dysfunction. Metabolic dysfunction is defined as the existence of at least two risk factors: (1) Waist circumference: ≥ 90 cm (male) and 80 cm (female); (2) Blood pressure: ≥ 130/85 mmHg or receiving treatment for lowering blood pressure; (3) Blood triglycerides: ≥ 1.7 mmol/L or receiving lipid-lowering drugs; (4) Plasma high-density lipoprotein (HDL) cholesterol: < 1.0 and 1.3 mmol/L for men and women, respectively, or receiving lipid-regulating drugs; (5) Pre-diabetes: fasting blood glucose 5.6–6.9 mmol/L or 2 h postprandial blood glucose 7.8–11.0 mmol/L or glycated hemoglobin of 5.7%–6.4%; (6) insulin resistance index assessed by the homeostasis model: ≥ 2.5; and (7) blood ultrasensitive C-reactive protein: ≥ 2 mg/L. Patients with contraindications to MRI and inability to hold their breath for >3 s during US examination were excluded. Demographic and clinical data were recorded for all participants. All participants underwent USFF, CAP, and ^1^H-MRS assessment on the same day or within 14 days when possible. The inclusion and exclusion criteria and study flow chart are shown in Fig. 1. No participants underwent any treatment prior to the imaging examination.

### B-mode ultrasound measurements

The R10 Prestige US system (Samsung Medison Co. Ltd., Hongcheon, Korea) was used to perform the abdominal scans using a CA 1–7 S convex probe (1–7 MHz). Other clinical information about the participants was kept confidential by the radiologist. Characteristics of fatty liver on the gray-scale US included parenchymal echogenicity enhancement, attenuation of far-field echoes, increased hepatorenal contrast, decreased visibility of the diaphragm line and intrahepatic portal vein wall, and decreased visibility of deep liver structures [25]. Subsequently, VHSG was assessed by a radiologist using gray-scale US images obtained during B-mode US examination of the liver parenchyma [26]. The scoring system is as follows: 0, no fatty liver; 1, mild fatty liver; 2, moderate fatty liver; and 3, severe fatty liver. Mild steatosis was defined as increased echogenicity of the liver compared with that of the right renal cortex. Moderate steatosis refers to hepatic echogenicity greater than that of the right renal cortex with insignificant perihepatic echogenicity. Severe steatosis was characterized by increased hepatic echogenicity that obscures diaphragmatic echogenicity.

### USFF data acquisition

During each examination, the radiologist performed at least five data acquisitions at the same location in the right hepatic lobe by using the lateral intercostal approach. Participants were positioned in a supine position with the right arm in maximum abduction. Prior to the first data acquisition, the system settings were adjusted for each participant to optimize visualization of the right lobe of the liver and to identify areas of liver parenchyma with localized fat deposits and large blood vessels to be avoided. The settings were kept constant for subsequent examinations. Each acquisition was performed by the operator pressing a button once to record a B-mode image and TAI and TSI values. Prior to each data acquisition, participants paused for breath after shallow inspiration. *R*^2^ values were used to optimize the accuracy of ROI. The operator’s goal is to obtain *R*^2^ values > 0.80 for all measurements. TAI is reported in dB/cm/MHz, while TSI is reported in arbitrary units [25]. After complete measurement of TAI and TSI values, the system automatically outputs the corresponding USFF values.

### CAP data acquisition

FibroScan 502 Touch (Echosens, Paris, France) was used for CAP measurements. All CAP measurements were performed using a standardized protocol [17] by a gastroenterologist with more than 5 years of experience, who was blinded to the other results. The scans were performed on the same day as the US. The subjects were placed in the supine position with the right arm maximally abducted, and measurements of the right hepatic lobe were made through the intercostal space. The selection of the same intercostal area for QUS measurements ensures that the measurement area is as consistent as possible. The rationale for the CAP has been illustrated elsewhere [17]. The CAP was taken ten times, and the median and IQR values were calculated and recorded.

### ^1^H-MRS data acquisition

Participants underwent ^1^H-MRS examinations using an Avanto 1.5 T MR scanner (Siemens, Erlangen, Germany) within 1 week before/after the US scan. Participants were examined in a supine position and underwent breathing exercises prior to the examination. To localize spectroscopically collected voxels, sagittal, coronal, and axial sections covering the entire liver were collected. A single 8 cubic centimeter (2 × 2 × 2 cm) voxel was placed within the right lobe, avoiding major vascular structures and subcutaneous adipose tissue. After shimming the body of interest, a proton spectrum was acquired using a body coil. Proton spectra were acquired using a point-resolved spectroscopy (PRESS) sequence with the following parameters: repetition time = 1500 ms, echo time = 135 ms. Signal intensities of the water peak at 4.8 ppm (Sw) and the fat peak at 1.4 ppm (Sf) were measured at 1.4 ppm and 1.4 ppm, respectively. The percentage of liver fat was calculated using the formula 100 × Sf/(Sf + Sw), where Sf and Sw represent the signal intensities of the fat peak at 1.4 ppm and the water peak at 4.8 ppm, respectively. All MRS analysis results were analyzed by radiologists whose information was inter-blinded. The guidelines recommend MRI-based techniques, such as MRI-PDFF and ^1^H-MRS, as the gold standard for quantifying liver fat. Therefore, we used MRS as the gold standard and classified hepatic steatosis into four grades based on previous studies: S0 (^1^H-MRS < 5.56%), S1 (^1^H-MRS = 5.56–12.7%), S2 (^1^H-MRS = 12.7–18.9%), and S3 (^1^H-MRS ≥ 18.9%) [34, 35].

### Statistical analysis

Continuous variables were expressed as means and standard deviations, and categorical variables were expressed as counts and percentages. Pearson correlation coefficients were calculated between USFF, CAP, and ^1^H-MRS for all participants. Bland-Altman analysis with 95% limits of agreement was used to test the correlation between USFF and MRS. Linear regression analysis was used to evaluate linear regression slope, intercept, and *R*^2^. A two-tailed *t*-test or Mann–Whitney *U-*test was performed for each quantitative USFF, CAP to determine whether the means of the USFF, CAP were statistically different between groups of participants. The significance level was set at *p* < 0.05. Receiver operating characteristic curve analysis (ROC) was used to compare the performance of USFF, CAP, VHSG in detecting varying degrees of hepatic steatosis of NAFLD. For each ROC analysis, the area under the ROC curve, sensitivity, specificity, positive and negative predictive values (NPVs) were calculated. AUCs were compared using the method described by DeLong et al [36]. All statistical analyses were performed using commercially available software (SPSS version 26.0; IBM Corporation, Armonk, NY, USA; and GraphPad Prism version 8.0; GraphPad Software Corporation, San Diego, CA, USA).

## Results

### Patient characteristics

In total, 113 prospectively recruited participants (mean age, 44.79 ± 13.56 years; 42 women (37.17%), 71 men (62.83%), mean BMI, 26.83 ± 5.57) with or suspected of having MAFLD, with both ^1^H-MRS, CAP and USFF measurements, were included in this study. The mean ^1^H-MRS was 16.45% ± 10.50% (range, 1.42%–57.70%). 98 (86.72%) of the participants had MAFLD (defined as ^1^H-MRS ≥ 5.56%) and 65 (57%) of the participants had ^1^H-MRS of 12.7% or more. The mean USFF was 18.81% ± 7.76% (range, 1.86%–46.60%). The mean CAP was 307.4 ± 51.51 dB/m (range, 175–400 dB/m). The baseline demographic, biochemical, histological, and imaging data of the participants were summarized in Table 1.

**Table 1:** Demographic, biochemical, histological, and imaging data of the participants

Variable	Value
Sex	Female: 42 (37.17%) Male: 71 (62.83%)
Age^a^	44.79 ± 13.56 (22–76)
BMI (kg/m^2^)^a^	26.83 ± 5.57
White blood cell (WBC 10^9^/L)^a^	6.79 ± 1.56
Neutrophil ratio (RGB %)^a^	152.00 ± 6.56
Platelets (PLT 10^9^/L)^a^	233.62 ± 59.60
Glutamic oxaloacetic transaminase (AST U/L)^a^	34.98 ± 29.37
Glutamic pyruvic transaminase (ALT U/L)^a^	51.40 ± 41.16
γ-glutamyl transpeptidase (γ-GGT U/L)^a^	57.35 ± 50.26
Alkaline phosphatase (ALP U/L)^a^	81.54 ± 23.54
Serum albumin (ALB g/L)^a^	49.08 ± 3.44
Serum creatinine (Cre μmol/L)^a^	79.26 ± 16.90
Blood urea nitrogen (BUN mmol/L)^a^	5.40 ± 1.17
Total bilirubin (TBil μmol/L)^a^	13.90 ± 6.09
Direct bilirubin (DBil μmol/L)^a^	3.67 ± 2.26
Triglycerides (TG mmol/L)^a^	2.09 ± 2.66
Total cholesterol (TC mmol/L)^a^	5.08 ± 1.27
High-density lipoprotein (HDL mmol/L)^a^	1.28 ± 0.48
Low-density lipoprotein (LDL mmol/L)^a^	3.15 ± 1.06
Skin-to-liver capsule distance (cm)^a^	2.18 ± 0.57
Proton magnetic resonance spectroscopy (^1^H-MRS)^a^	16.45% ± 10.50
Quantitative ultrasound system fat fraction (USFF)^a^	18.81% ± 7.76%
Controlled attenuation parameter (CAP)^a^	307.4 ± 51.51

All laboratory examinations were performed while patients were fasting

^a^Mean with a standard deviation

### Correlation between USFF and ^1^H-MRS

**Fig. 2:**
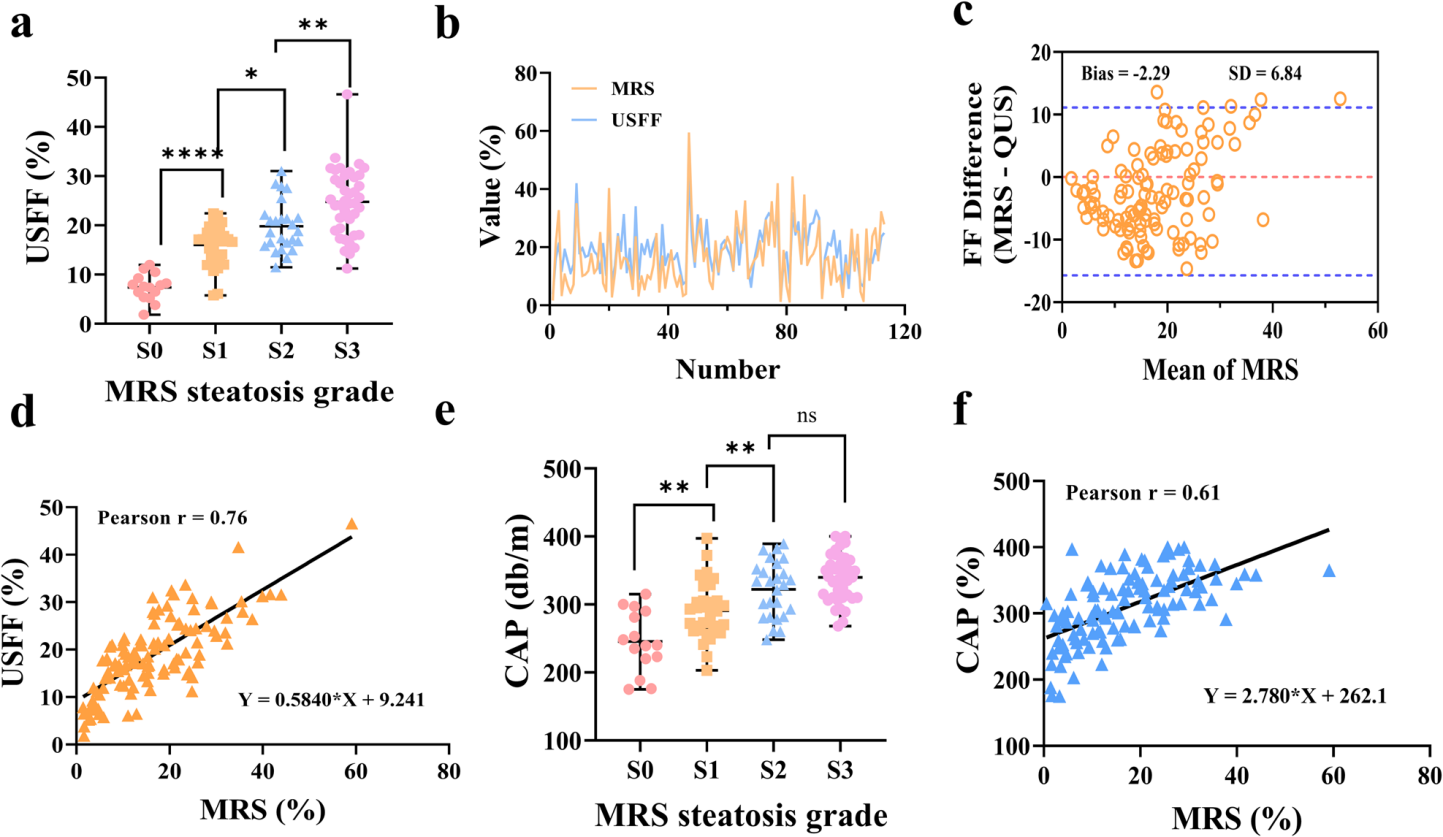
Correlation of USFF with ^1^H-MRS and CAP with ^1^H-MRS. **a** The degree of hepatic steatosis was graded according to the ^1^H-MRS results, and the distribution of USFF in different grades. *t*-test: S1-S0: *p* < 0.0001; *t*-test: S2-S1: *p* = 0.0024; *t*-test: S3-S2: *p* = 0.0028. **b** USFF values and ^1^H-MRS values corresponding to each participant. **c** Bland-Altman plot shows the difference between USFF and the ^1^H-MRS. **d** USFF vs. ^1^H-MRS scatterplot, Person correlation analysis, and linear regression line. **e** The degree of hepatic steatosis was graded according to the ^1^H-MRS results, and the distribution of CAP in different grades. *t-*test: S1-S0: *p* < 0.0012; *t*-test: S2-S1: *p* = 0.0057; *t*-test: S3-S2: *p* = 0.0687. **f** CAP vs. ^1^H-MRS scatterplot, Person correlation analysis, and the linear regression line

The scatterplot in Fig. 2a, b illustrates the distribution of USFF values according to the grade of liver steatosis. The mean USFF values of all participants with ^1^H-MRS = S1 (16.00% ± 4.08%; range, 5.79%–22.44%) were significantly higher than those of controls without steatosis (7.42% ± 2.73%; range, 1.86%–11.98%; *p* < 0.0001). In addition, participants with steatosis with ^1^H-MRS = S3 had significantly higher USFF values (24.77% ± 6.86%; range, 11.23%–46.60%, *p* < 0.0028) compared to other participants in the hepatic steatosis group (Fig. 2a, b and Table 2).

**Table 2:** The hepatic fat content of all participants was graded based on ^1^H-MRS results

Variable	Grade	Total patients (*n* = 113)	Mean ± SD
^1^H-MRS	S0	15 (13.27%)	3.22 ± 1.22
	S1	33 (29.20%)	9.21 ± 2.24
	S2	25 (22.12%)	15.72 ± 1.83
	S3	40 (35.40%)	27.82 ± 8.07
USFF	S0	15 (13.27%)	7.42 ± 2.73
	S1	33 (29.20%)	16.00 ± 4.08
	S2	25 (22.12%)	19.82 ± 5.09
	S3	40 (35.40%)	24.77 ± 6.86
CAP	S0	15 (13.27%)	245.3 ± 44.88
	S1	33 (29.20%)	291.0 ± 41.51
	S2	25 (22.12%)	322.1 ± 41.03
	S3	40 (35.40%)	339.8 ± 35.40

The mean ± SD of ^1^H-MRS, USFF, and CAP were calculated across the different grades measured

*TAI* tissue attenuation imaging, *TSI* tissue scatter distribution imaging, *USFF* quantitative US (QUS) fat fraction (USFF), ^*1*^*H-MRS* proton magnetic resonance spectroscopy, *CAP* controlled attenuation parameter. ^1^H-MRS: S0-S1: *p* < 0.001, S2-S1 *p* < 0.001, *p* < 0.001; USFF: S0-S1 *p* < 0.001, S2-S1 *p* = 0.0024, *p* = 0.0028; CAP: S0-S1 *p* < 0.0012, S2-S1 *p* = 0.0057, *p* = 0.0687

The agreement between USFF and MRS is shown on Bland-Altman plots and scatter plots (Fig. 2c). The mean deviation between USFF and ^1^H-MRS was −2.29 ± 6.84%. The 95% coincidence range was −15.71% and 11.12%. The USFF showed a strong correlation with the ^1^H-MRS (*r* = 0.76, 95% confidence interval (CI): 0.82, 0.90; *p* < 0.001). In the linear range, linear regression analysis of USFF versus ^1^H-MRS showed a slope of 0.58 (95% CI: 0.49, 0.68), an intercept of 9.24 (95% CI: 7.40, 11.08), and an *R*^2^ of 0.58 (Fig. 2d).

### Correlation between CAP and ^1^H-MRS

The distribution of CAP in different grades of hepatic steatosis was shown in Fig. 2e, and the results indicated that CAP was significantly different in varying degrees of hepatic steatosis. In addition, CAP was correlated with ^1^H-MRS (*r* = 0.61, 95% CI: 0.48, 0.71; *p* < 0.001). However, the correlation was poorer compared with USFF. CAP had some linear relationship with MRS. Within the linear range, linear regression analysis of USFF with ^1^H-MRS showed a slope of 2.78 (95% CI: 0.56, 0.85), an intercept of 262.1 (95% CI: −0.11, 1.63), and an *R*^2^ of 0.37. However, when the ^1^H-MRS ≥ 20%, the CAP tended to saturate without a significant linear relationship (Fig. 2f).

### Comparison between USFF, CAP, and VHSG in the detection of ^1^H-MRS ≥ S1

**Fig. 3:**
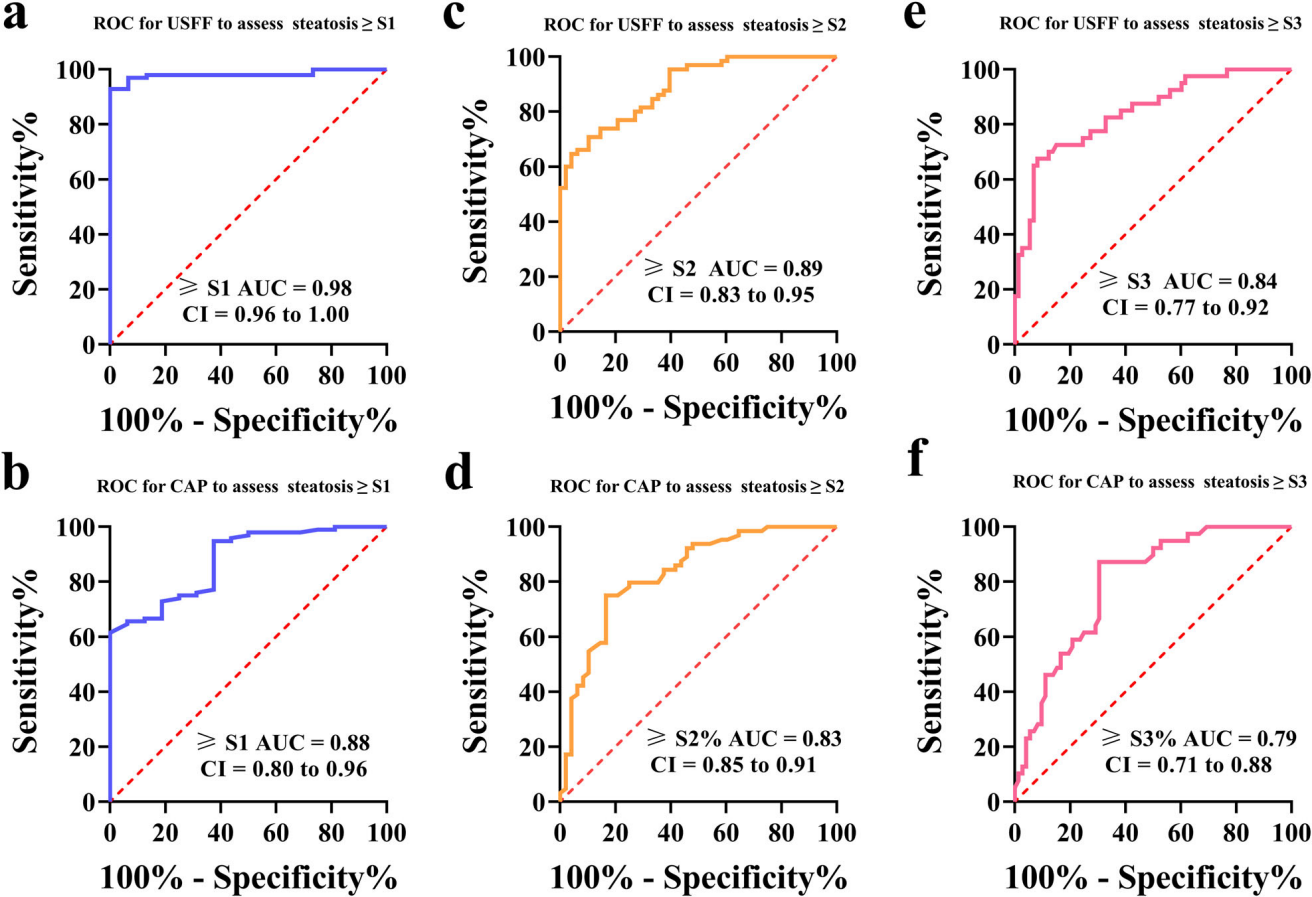
Comparison of the performance of USFF and CAP in detecting different grades of steatosis. **a** Receiver operating characteristic curves used to detect ≥ S1 (^1^H-MRS ≥ 5.56%) hepatic steatosis for USFF. **b** Receiver operating characteristic curves used to detect ≥ S1 (^1^H-MRS ≥ 5.56%) hepatic steatosis for CAP. **c** Receiver operating characteristic curves used to detect ≥ S2 (^1^H-MRS ≥ 12.7%) hepatic steatosis for USFF. **d** Receiver operating characteristic curves used to detect ≥ S2 (^1^H-MRS ≥ 12.7%) hepatic steatosis for CAP. **e** Receiver operating characteristic curves used to detect ≥S3 (^1^H-MRS ≥ 18.9) hepatic steatosis for USFF. **f** Receiver operating characteristic curves used to detect ≥ S3 (^1^H-MRS ≥ 18.9) hepatic steatosis for CAP. Pairwise comparison of receiver operating characteristic curves: ≥ S1: USFF-CAP: *p* = 0.024; USFF-VHSG: *p* = 0.004; CAP-VHSG: *p* = 0.190; ≥ S2: USFF-CAP: *p* = 0.231; USFF-VHSG: *p* < 0.001; CAP-VHSG: *p* = 0.001; ≥S3: USFF-CAP: *p* = 0.433; USFF-VHSG: *p* = 0.004; CAP-VHSG: *p* = 0.020

Table 3 summarizes the performance of assessing steatosis by USFF, CAP, and VHSG based on ^1^H-MRS. The AUCs of USFF, CAP, and VHSG used to assess steatosis based on ^1^H-MRS ≥ S1 were 0.98 (95% CI: 0.96–1.00), 0.88 (95% CI: 0.80–0.96) and 0.85 (95% CI: 0.75–0.95), respectively (Fig. 3a, b and Table 3). In the receiver operating characteristic analysis, USFF had the highest diagnostic performance with a cut-off value of 12.01% and sensitivity and specificity of 92.86% and 100% respectively. In addition, CAP had the highest diagnostic performance with a cut-off value of 254.5 dB/m and sensitivity, and specificity of 94.79% and 62.50%, respectively. Also, hepatic steatosis was present when the VHSG ≥ 1, with sensitivity, and specificity of 98.98%, and 46.67%, respectively. When assessing hepatic steatosis based on ^1^H-MRS ≥ S1, USFF had significantly higher diagnostic performance than CAP and VHSG (*p* = 0.024 and *p* = 0.004, respectively).

**Table 3:** Comparison of the diagnostic performance between USFF, CAP, and VHSG in the detection of varying degrees of hepatic steatosis

Variables	Cut-off value	Sensitivity (%)	Specificity (%)	Accuracy (%)	PPV (%)	NPV (%)	AUC (95% CI)
≥ S1							
USFF	12.01	92.86	100.00	93.81	100.00	68.18	0.98 (0.96–1.00)
CAP	254.5	94.79	62.50	88.50	91.84	66.67	0.88 (0.80–0.96)
VHSG	≥ 1	98.98	46.67	92.92	92.38	87.50	0.85 (0.75–0.95)
≥ S2							
USFF	19.98	64.62	95.83	76.11	91.49	65.15	0.89 (0.83–0.95)
CAP	277.0	92.19	54.17	76.99	73.81	76.99	0.83 (0.75–0.91)
VHSG	≥ 2	62.12	68.09	64.61	73.21	56.14	0.67 (0.58–0.78)
≥ S3							
USFF	22.22	67.50	91.78	83.19	82.35	83.54	0.84 (0.77–0.92)
CAP	305.5	87.18	69.44	81.41	69.49	94.44	0.79 (0.71–0.88)
VHSG	≥ 3	25.00	89.04	66.37	55.56	68.42	0.72 (0.63–0.82)

*USFF* quantitative US (QUS) fat fraction (USFF), ^*1*^*H-MRS* proton magnetic resonance spectroscopy, *CAP* controlled attenuation parameter, *VHSG* visual hepatic steatosis grade, *AUC* area under the receiver operating characteristic curve, *NPV* negative predictive value, *PPV* positive predictive value. Pairwise comparison of receiver operating characteristic curves:

≥ S1: USFF-CAP: *p* = 0.024; USFF-VHSG: *p* = 0.004; CAP-VHSG: *p* = 0.190;

≥ S2: USFF-CAP: *p* = 0.231; USFF-VHSG: *p* < 0.001; CAP-VHSG: *p* = 0.001;

≥ S3: USFF-CAP: *p* = 0.433; USFF-VHSG: *p* = 0.004; CAP-VHSG: *p* = 0.020

### Comparison between USFF, CAP, and visual score in the detection of ^1^H-MRS ≥ S2

The AUCs of USFF, CAP, and visual scores for the detection of hepatic steatosis (^1^H-MRS ≥ S2) were 0.89 (95% CI: 0.83–0.95), 0.85 (95% CI: 0.85–0.91), and 0.67 (0.58–0.78). The cut-off values for the detection of hepatic steatosis (^1^H-MRS ≥ S2) were USFF of 19.98%, CAP of 277 dB/m, and VHSG of 2, respectively (the corresponding sensitivity, and specificity are shown in Fig. 3c, d and Table 3). When assessing hepatic steatosis based on ^1^H-MRS ≥ S2, USFF had significantly higher diagnostic performance than VHSG (*p* < 0.001).

### Comparison between USFF, CAP, and visual score in the detection of ^1^H-MRS ≥ S3

The study found that for ^1^H-MRS ≥ S3 the diagnostic performance of USFF was not significantly different from that of CAP (*p* = 0.433), but was significantly higher than that of VHSG (*p* = 0.004). The AUC for USFF was 0.84, while for CAP and VHSG it was 0.79 and 0.72, respectively (Fig. 3e, f and Table 3). When the cut-off value of USFF was 22.22%, the diagnostic sensitivity and specificity were 67.50% and 95.83%, respectively. When the cut-off value of CAP was 305.5 dB/m, its sensitivity and specificity were 87.18% and 69.44%, respectively. When the cut-off value of VHSG was 3, its sensitivity and specificity were 25% and 89%, respectively.

## Discussion

MAFLD has become the most common liver disease and a significant cause of liver disability and death worldwide [33]. Therefore, it is extremely important to discern and accurately quantify the extent of hepatic steatosis and fat content during the initial stage [31]. Therefore, we sought to investigate the correlation between the USFF technique and ^1^H-MRS in a well-characterized prospective cross-sectional observational study of participants with or suspected of having MAFLD, along with a comparison of different imaging modalities. The study found that the USFF technique correlates well with ^1^H-MRS and was superior to other imaging techniques in identifying various grades of hepatic steatosis. This provides a cost-effective and convenient method for identifying, monitoring, and predicting outcomes for patients with MAFLD.

Related research has discovered that both CAP and USFF are capable of quantitatively assessing hepatic steatosis and are directly correlated with the severity of hepatic steatosis [25, 31]. Nevertheless, their utilization of distinct imaging principles and calculation methodologies results in varying quantitative parameters and diagnostic efficacy. Recognizing these discrepancies is crucial for both clinical practice and research purposes. Numerically, USFF showed a good correlation with ^1^H-MRS (*r* = 0.76). The most widely used US attenuation technique, CAP, had a moderate correlation coefficient with ^1^H-MRS (*r* = 0.61), similar to previous studies [37]. Comparing with CAP, USFF had a better correlation and demonstrated a better linear relationship with ^1^H-MRS. However, when MRI ^1^H-MRS ≥ 20%, CAP showed a saturation effect and hence had a poorer linear relationship with ^1^H-MRS. This suggested that the USFF technique was more accurate than CAP in higher grades of steatosis, and therefore, further comparison of their diagnostic performance was extremely important.

Our results showed that USFF had excellent diagnostic performance (AUC = 0.98) for diagnosing hepatic steatosis (^1^H-MRS ≥ S1). It was significantly higher than the CAP and subjective visual scores (0.88 and 0.85; *p* = 0.024 and *p* = 0.004, respectively). Given its excellent diagnostic performance, USFF was expected to be a noninvasive and accurate assessment tool for liver fat quantification in patients with MAFLD. It was also expected to be a potential diagnostic enrichment biomarker for identifying patients who could benefit from drug trials. High assessment failure rates have been reported in MAFLD-related drug trials due to participants not meeting MRI-derived PDFF criteria, regardless of their preselected CAP results [38]. USFF had a positive predictive value (PPV) of ≥ S1 (100%) for the diagnosis, which may help to reduce assessment failure rates in MAFLD-related drug trials.

In addition, previous studies have not explored and compared the performance of USFF and CAP for the diagnosis of moderate-to-severe hepatic steatosis (^1^H-MRS ≥ S2 and ^1^H-MRS ≥ S2). Early identification of moderate-to-severe grade steatosis was more clinically valuable, contributing to the recognition and early intervention of metabolic-associated steatohepatitis (MASH) and to the assessment of the efficacy of related treatments. The results showed that USFF had a good diagnostic performance (AUC = 0.89). It was significantly higher than the VHSG (0.85 and 0.67, respectively; *p* < 0.001 and *p* = 0.004). At the same time, its performance was not inferior to CAP for recognizing moderate to severe steatosis. Meanwhile, although the optimal cut point for CAP to diagnose MAFLD varied, USFF was directly correlated with the ^1^H-MRS results and provided a more intuitive response to the degree of hepatic steatosis. This suggested that the USFF technique was an efficient, convenient, and highly accurate tool for the early recognition of MASH. This study also provided reference cut-off values for different grades of hepatic steatosis, which were 12.01%, 19.98%, and 22.22%, corresponding to S1, S2, and S3, respectively.

Our study had several limitations. First, participants were skewed toward steatosis; the proportion of participants without steatosis was 13.3% (15 of 113), which does not characterize the distribution of real-world populations and may have contributed to the high cut-off value for the diagnosis of hepatic steatosis (^1^H-MRS ≥ 5.56%). To improve the accuracy of our diagnostic thresholds, it is necessary to include additional healthy volunteers without steatosis. Second, our study was a single-center study with a study population skewed toward mild and moderate steatosis, which may have led to some selection bias. Further multicenter validation is needed. In addition, due to the small sample size, this study failed to assess potential confounders such as inflammation or fibrosis, thus more studies are needed in the future. Finally, compared to MRI-PDFF, ^1^H-MRS can only analyze a portion of the liver parenchyma, while MRI-PDFF can scan and analyze the entire organ. Therefore, MRS is susceptible to specimen error. Additionally, ^1^H-MRS can only analyze the fat content of liver parenchyma and does not reflect the degree of inflammation and fibrosis of the liver, unlike liver biopsy. Therefore, in future studies, we will investigate the role of multiparametric ultrasound in identifying fibrosis and inflammation using liver biopsy as the gold standard. Meanwhile, USFF is similar to ^1^H-MRS as it analyses a portion of the liver parenchyma rather than the whole organ and therefore has some sampling error. It is also susceptible to respiratory effects, and some older patients may be unable to hold their breath well, resulting in measurement errors.

In conclusion, our study has demonstrated that quantitative USFF offers a strong correlation with ^1^H-MRS, enabling more accurate identification of hepatic steatosis than both CAP and VHSG, and is expected to be a valuable tool in the assessment of MAFLD and MASH.

## Data Availability

The datasets used or analyzed during the current study are available from the corresponding author upon reasonable request.

## Abbreviations

^1^H-MRS: Proton magnetic resonance spectroscopy
AUC: Area under the curve
BMI: Body mass index
CAP: Controlled attenuation parameter
CI: Confidence interval
MAFLD: Metabolic-associated fatty liver disease
NPV: Negative predictive value
PPV: Positive predictive value
QUS: Quantitative ultrasound
ROC: Area under the receiver operating characteristic curve
TAI: Tissue attenuation imaging
TSI: Tissue scatter distribution imaging
USFF: Quantitative ultrasound system fat fraction
VHSG: Visual hepatic steatosis grade
